# CRT Pacing: Midterm Follow-Up in LV Only Pacing without RV Lead in Patients with Normal AV Conduction

**DOI:** 10.3390/jcm7120531

**Published:** 2018-12-08

**Authors:** Dragos Cozma, Cristina Vacarescu, Lucian Petrescu, Cristian Mornos, Emilia Goanta, Horea Feier, Constantin Tudor Luca, Gabriel Gusetu, Radu Vatasescu

**Affiliations:** 1 University of Medicine and Pharmacy “Victor Babes”, 300310 Timisoara, Romania; dragoscozma@gmail.com (D.C.); vacarescucristina@yahoo.com (C.V.); petrescu_lucian@yahoo.com (L.P.); horea.feier@gmail.com (H.F.); costiluca67@yahoo.ro (C.T.L.); 2 Institute of Cardiovascular Diseases, 300310 Timisoara, Romania; ema.goanta@yahoo.com; 3 University of Medicine and Pharmacy “Iuliu Hațieganu”, 400347 Cluj, Romania; gusetu@gmail.com; 4 Cardiology Department, Clinical Rehabilitation Hospital, 400347 Cluj, Romania; 5 University of Medicine and Pharmacy “Carol Davila”, 014451 Bucharest, Romania; radu_vatasescu@yahoo.com; 6 Clinical Emergency Hospital, 014451 Bucharest, Romania

**Keywords:** cardiac resynchronization therapy, right atrium/left ventricular pacing, normal atrio-ventricular conduction

## Abstract

Background: The aim of our study was to assess the real life cardiac resynchronization therapy (CRT) fusion left ventricular (LV) only pacing in patients with normal AV conduction (NAVc) without right ventricular (RV) lead. Methods: Consecutive NAVc patients with CRT indication were implanted with a right atrium RA/LV DDD pacing system. Complete follow-up at 1, 3 and every 6 months thereafter included echocardiography and stress testing. Results: We analysed 55 patients (62 ± 11 years). All patients were responders with significant LV reverse remodelling (LV end-diastolic volume 193.7 ± 81 vs. 243.2 ± 82 mL at baseline, *p* < 0.002) and increased LV ejection fraction (38 ± 7.9% vs. 27 ± 5.2% at baseline, *p* < 0.001). Mitral regurgitation decreased in 38 patients (69%). During follow-up (35 ± 18 months), 20 patients (36%) needed reprogramming sensed/paced AV delay or maximum tracking rate (MTR) because of inadequate or lost LV capture at exercise test; personalized programming to achieve up to 100% fusion pacing was used in all patients. One patient developed Mobitz II second degree AV block and triple chamber CRT-P upgrade was performed; defibrillator upgrade was not necessary. Conclusions: LV only pacing CRT-P without RV lead showed a positive outcome in carefully selected patients.

## 1. Introduction

Cardiac resynchronization therapy (CRT) currently involves a mandatory implantation of right ventricular (RV) lead in the setting of biventricular pacing. Intrinsic activation of the left ventricle (LV) is judged to be better than activation through RV pacing. RV pacing is known to deteriorate RV/LV function and should be avoided in patients with sinus rhythm and normal atrio-ventricular (AV) conduction. However the guidelines [1] allows simultaneous biventricular pacing with 0 ms VV interval. LV only pacing has been proposed several years ago and tested in acute [2], in chronic studies [3,4] and the extended available data and results permitted the introduction of LV only stimulation concept in the current guideline. Despite noninferiority to biventricular pacing [4,5], it is not widely used in clinical practice. Moreover, it was of limited use before Adaptive CRT (aCRT) trial [6]. The explanation is mainly due to increase of LV threshold or inadequate percentage of pacing (but this may obvious affect classic CRT pacing). The main criticism for not using RV lead would be AV block occurrence and variability of AV conduction, therefore there are very limited available data concerning LV pacing without RV lead for resynchronization [7,8].

On the other hand a RV lead is mandatory in CRT-defibrillator (CRT-D), which is required in secondary prevention and would be more appropriate in younger patients with ischemic heart disease, according to the guidelines [1]. Subsequently, the only need to implant a RV lead for CRT in post Danish study [9] era may be that of CRT-D, AV block, as well as in patients with permanent atrial fibrillation (PAF), in whom RA-triggered LV pacing cannot be done. 

The hypothesis of this study is founded on the fact that in patients with heart failure (HF), systolic dysfunction, left bundle branch block (LBBB) and preserved AV conduction, intrinsic depolarization of the ventricles is better than RV pacing (DAVID trial) [10], while they need LV delayed activation to be corrected through LV pacing. Our assumption was that promoting only LV stimulation without the possibility of interfering RV stimulation will lead to a positive outcome. The purpose of this study is to assess outcome of real life CRT fusion pacing using only right atrium/left ventricular (RA/LV) leads in patients with normal AV conduction (NAVc) without coronary artery disease. 

## 2. Experimental Section

This prospective study was conducted in 3 CRT centres in Romania: Timisoara Institute of Cardiovascular Diseases (42 pts), Clinical Emergency Hospital Bucharest (9 pts) and Clinical Rehabilitation Hospital—Cardiology Department, Cluj (4 pts). Consecutive class II-III NYHA HF pts with CRT-P indication, LBBB morphology and NAVc (PR interval < 250 ms), without significant coronary artery disease were implanted with a RA/LV DDD pacing system. The exclusion criteria were represented by PAF, syncope, history of myocardial infarction, Wenkebach point < 120/min (>500 ms), CRT-D indication in secondary prevention, severe morbidity (severe hepatic or renal failure, pulmonary, cerebral). Patients judged to be implanted with CRT-D (patients choice or doctor choice) were also excluded. 

Transthoracic echocardiography (TTE) was performed in all patients. Ultrasound images were explored with patients in the left lateral decubitus position, using a VIVID 9 system (GE Health Medical, Milwaukee, WI, USA). ECG was simultaneously recorded for each patient. The echocardiographic examination was performed using standard views and techniques. LV wall thickness, LV end-diastolic and end-systolic diameters (LVEDD, LVESD) and volumes (LVEDV, LVESV), LV ejection fraction (LVEF), LA diameter (LAd), LA area (LAA) and LA volume (LAV) were measured. Atrioventricular, interventricular, intra-LV synchrony parameters were also assessed in all patients. All LA measurements were performed at end-systole just before mitral valve opening and maximal atrium size was considered for evaluation. Complete TTE evaluation for CRT was performed in all patients [11].

**Figure 1:**
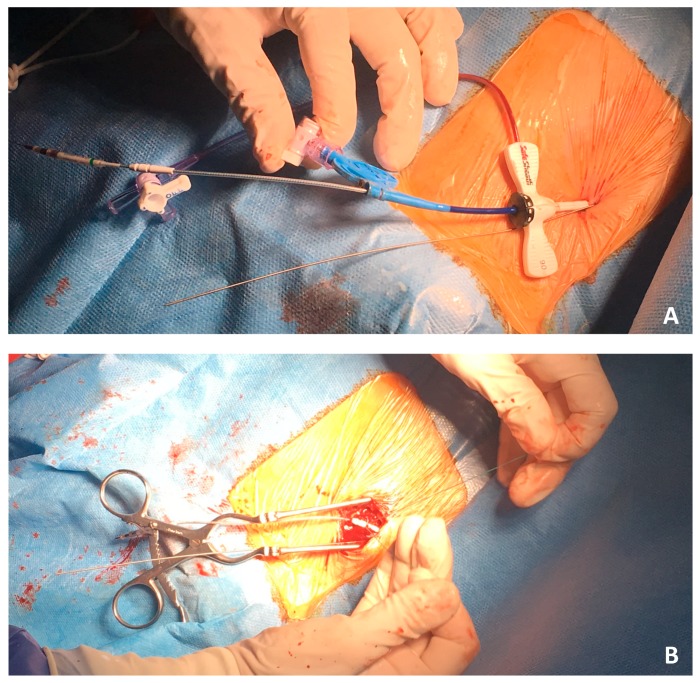
(**A**) direct percutaneous subclavian vein puncture with left ventricular lead placement in coronary sinus target vein followed by sheath removal; (**B**) incision and left ventricular lead fixation.

Implantation strategy: direct percutaneous subclavian vein puncture was performed to the skin without any incision (Figure 1A) unless successful LV lead placement in coronary sinus target vein: posterior, posterolateral, lateral or anterolateral. The following step was sheath removal, incision and LV lead fixation (Figure 1B). Pocket dissection for pacemaker was performed and finally RA lead placement. In the case of any AV block occurrence during implantation, a backup plan was ready with RV lead placement and true triple chamber CRT-P available, without incision unless LV placement likewise. In patients not feasible for endocardial pacing due to unfavourable coronary sinus anatomy, everything was withdraw, no implantation was done in the first step; if a second attempt was unsuccessful, then epicardial LV lead was planned and placed using minithoracotomy with subsequent implantation of a dual chamber pacemaker using direct subclavian vein puncture for RA lead.

The device was interrogated and intuitively programmed immediately after procedure to achieve fusion. After 24 h next to the implantation following steps were performed: complete device interrogation, 12 ECG leads pacing on/off, TTE on/off; final best device programming using both ECG and TTE. The purpose was to obtain the best compromise between longest diastolic filling, presence of R wave in V1, q or Q wave in V6 on ECG and best echocardiographic parameters (AV, interventricular, intra-LV synchrony parameters). The AV interval was programmed individually to obtain the best fusion capture and the stability of PR spontaneous interval was obtain by betablocker versus ivabradine dose titration in the manner that shorter PR interval patients permitted increased dosage of betablocker (BB) alone while longer PR interval patients were provided with ivabradine without BB or combination of lower dose BB.

Complete follow-up at 1, 3 and every 6 months thereafter included echocardiography, holter recording and exercise test (ET). The purpose was to quantify exercise functional capacity and to detect rhythm changes such as loss of ventricular capture, sinus tachycardia above upper tracking limit or shortening/lengthening of the intrinsic PR interval during ET. A cycloergometer ET was performed using a Bruce protocol, with an increase workload by 25 W for each 3 min exercise stage. A 12 leads ECG, METS and blood pressure were permanently recorded. 

At each follow-up the patients were checked for percentage of LV pacing (device interrogation data), LV threshold, spontaneous AV interval, capture quality using 12 ECG leads, review of pharmacological therapy, adequate response of pacemaker functioning to exercise (including AV interval or rate response adapted to the patient), percentage of pacing, 12 ECG leads pacing on/off and TTE on/off.

Criteria for responder patients [11,12] included the clinical CRT response (defined as improvement in NYHA class, ET workload and duration) and echocardiografic response (estimated by an increase in 5% LVEF or decrease by ≥15% in LVESV/LVEDV and decrease in mitral regurgitation severity). 

All subjects gave their informed consent for inclusion before they participated in the study. The study was conducted in accordance with the Declaration of Helsinki and the protocol was approved by the Ethics Committee of 1622/26.03.2014.

### Statistical Analysis 

Data are expressed as mean ± standard deviation for continuous variables and as proportions for categorical variables. Continuous variables were compared between groups using unpaired *t* test (variables with normal distribution) or Mann-Whitney U test (non-normally distributed variables). Proportions were compared using chi-square test and Fischer’s exact test. A *p* value < 0.05 was considered significant. All analyses were carried out with the SPSS, version 18.0 (SPSS Inc., Chicago, IL, USA) statistical software.

## 3. Results

**Figure 2:**
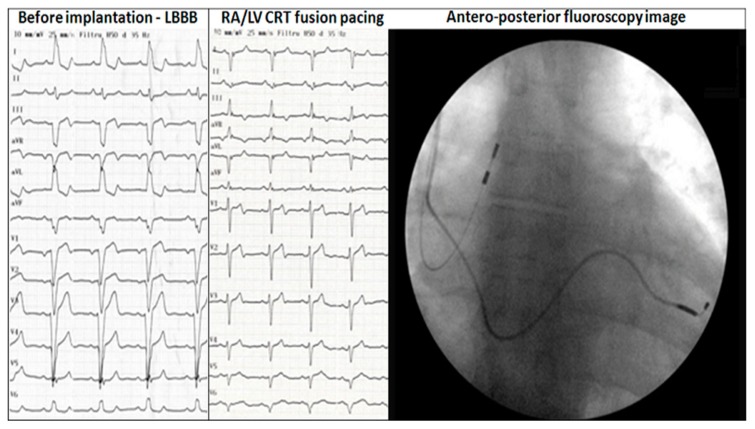
RA/LV DDD CRT in a 7 year follow up patient. ECG before and after cardiac resynchronization therapy: left bundle branch block (LBBB) pattern before implantation and antero-posterior fluoroscopy image; narrow QRS after implantation with negative QRS waves in DI, aVL, negative QRS waves in V6 and rS pattern in V1.

Fifty seven patients with idiopathic dilated cardiomyopathy and CRT-P indication were initially included. During implantation 2 patients developed acute AV block due to sheath manipulation (for one of them even temporary pacing was the choice of intraprocedural next step); the implantation was undergone with LV lead placement first. In these 2 patients AV block occurrence during implantation was followed by RV lead placement and true triple chamber CRT-P. Though these two patients were implanted with triple chamber CRT and excluded from the study, CRT fusion pacing RA/LV was used and AV block did not reoccurred in their follow-up. The final true RA/LV CRT-P group had 55 patients (Figure 2). Baseline demographic data are presented in Table 1. The average PR interval was 187 ± 32 ms (22 patients with PR between 200 and 250 ms). All patients had QRS complex >130 ms, LBBB morphology with a mean QRS interval of 164 ± 18 ms and a QRS axis of −17 (±36) degree. Baseline echocardiographic parameters are presented in Table 2.

All patients were implanted with a RA/LV DDD pacing system (8 patients—Biotronik, 8 patients—Medtronic, 34 patients—St. Jude Medical, 2 patients—Boston Stientific, 3 patients—Sorin Group). LV lead position was postero-lateral in 21 patients, lateral 19 patients, posterior 5 patients, anterolateral 7 patients, epicardial leads 3 patients. At baseline, all devices were programmed at a rest rate 60 beats/min and a maximum tracking rate (MTR) of 130 b/min. Individualized AV interval programming with an AV paced of 147 ± 22 ms and an AV sensed of 119 ± 25 ms, allowed fusion pacing in all patients. 

Post implantation ECG showed negative QRS or q waves in DI, aVL in 48 patients (87%) and negative QRS or q waves in V6 in 40 patients (73%). Rs pattern in V1 was noted in 32 patients (58%), while 22 patients (40%) had a rS aspect. 

Average follow-up was 35 ± 18 months. Mitral regurgitation decreased in 38 patients (69%). Echocardiographic asynchronism parameters improvement was observed in all patients. All patients were responders with LV reverse remodelling (LV end-diastolic volume = 193.7 ± 81 vs. 243.2 ± 82 mL at baseline, *p* < 0.002) and increased LV ejection fraction (38 ± 7.9% vs. 27 ± 5.2% at baseline, *p* < 0.001) (Table 3). 

One patient developed Mobitz II second degree AV block after exactly 3 years follow-up with alternating fusion narrow QRS and wide complete LV capture QRS. This 60 year-old female was under treatment with amiodarone/metoprolol for paroxysmal AF. The AV block persisted after amiodarone cessation and she was upgraded to a triple chamber CRT classic device, without complications. CRT-D upgrade was not necessary in any of our patients. 

The average percentage of pacing using device interrogation only in our group was 92 ± 6%. Although the quality of pacing (true fusion) could not be confirmed using only device statistics, ECG Holter and exercise test was used in all patients. Close attention and repetitive tests were used in patients with less than 95% ventricular pacing in order to ensure correct programming. 

ET using Bruce protocol was performed for all patients with a mean of 118 ± 41 Watts (6.7 ± 1.5 METS) during the follow-up period and a peak heart rate of 109 ± 13 b/min. During follow-up 20 patients (36%) needed reprogramming rate modulation parameters (DDDR mode) sensed/paced AV delay or maximum tracking rate (MTR) because of inadequate or lost LV capture at exercise test; personalized programming to achieve 100% fusion pacing was used in all patients. Post exercise test CRT optimization was performed by increasing MTR at 145 b/min in 6 patients and reprogramming rate adaptive AV interval in 15 patients. The dynamic AV interval was progressively decreased by 20 ms to ensure adequate and constant fusion pacing and a beat to beat ECG analysis was performed during all stages of exercise. Post ET optimization we registered a decrease in the AV interval by 23 ± 8 ms. Chronotropic incompetence was noted in 5 patients (9%) and rate modulation response was programmed after performing ET. 

Non-sudden cardiac death occurred in 5 patients (9%)**.** Readmissions due to worsening heart failure were noted in 17 patients (27%): 5 patients due to atrial flutter or fibrillation, 12 patients due to medication/diet issues. Pacemaker replacement was performed in 6 patients due to elective replacement indicator. 

Amiodarone was introduced for paroxysmal AF episodes (diagnosed at device interrogation) in 4 patients (7%). Direct current cardioversion for persistent AF was needed in 4 patients (7%). Typical atrial flutter cavotricuspid ablation was performed in another patient (2%). The final outcome was positive without any other complication in these patients. No evidence of recurrence of AV block was noted in those 2 patients who needed classic 3 leads CRT-P and LV only pacing was also the best strategy for long term follow-up.

BB and ivabradine titration was performed in 160 follow-ups from a total of over 370 recorded follow-ups. 

## 4. Discussion

This study presents new real life information regarding CRT without using RV lead; the outcome is positive for a population with mid-term follow-up of permanent and true LV only pacing in patients with NAVc. The data was widely debated and validated in the literature for LV only pacing noninferiority [13], however not without RV back-up pacing. To the best of our knowledge this is the largest group of CRT patients without using RV lead. In addition, the study is confirming the need of a complex and complete (including TTE and ET) survey of the CRT patients in order to maximize the percentage of responders. Changes in medication and reprogramming of the device during follow-up are of the utmost importance for the outcome. ET check was performed systematically in our series, with important implication for device reprogramming.

The AV conduction “normalization” during effort may lead to decrease of LV stimulation and this may be the main criticism of this study as we did not use any automatic algorithm to avoid under LV fusion pacing except strict controls, ET and manual programmed AV interval. The most important algorithm for LV only stimulation is describe by aCRT trial investigators [6] in 428 randomized patients as a safe, effective algorithm and new standard which promotes intrinsic conduction and reduces RV pacing in heart rate <100/min but not over this heart rate. This value was intuitively and empirically chose without evidence based. However, upper limit of the LV only pacing in the adaptive CRT algorithm was set based on the hemodynamic study [14] showing the superiority of biventricular pacing over LV only pacing when heart rate increased >100/min. This study has important limitations: only 22 patients included (half ischemic aetiology) in acute study (DDD stimulated at 100/120/140/min) using invasive catheterisation, this was a non-real life situation short term outcome (our study is a real life midterm follow-up with exercise test).

Interestingly, in our study no difference in echocardiographic response and exercise test outcomes was observed these patients who needed reprogramming, may be due to high percentage overall LV pacing data showed initially at device interrogation. Holter monitoring were not helpful compared to exercise test in reprogramming devices in order to obtain better fusion pacing (time consuming repetitive Holter monitoring versus exercise test to obtain immediate results). 

LV only pacing on the other hand should be judged superior while exercising at a heart rate over 100/min; patients exercising at a higher heart rate with a rapid deceleration [15] correlates to responders; the need of LV only stimulation may prove to be the best choice in case of higher heart rate during exercise. On the other hand adaptive CRT is not available for AV interval longer than 200 ms. In our study we included on purpose patients with PR interval less than 250 ms; the possibility of LV pacing in patients with “moderate” first degree AV block using ivabradine for HF may allow simpler programming of the device and no loss of LV capture at ET occurred in our patients. Adaptive CRT reduced AF by 46% (control 16.2% vs. Adaptive CRT 8.7%) and hospitalization. Interesting the incidence of AF was 7% in our study population similar to a CRT trial [6].

There might be of critical concern the failure pf providing synchronous LV activation in case of AF occurrence but we still emphasize that the mere existence of RV lead does not ensure better outcome and does not change this concern, excepted cases with FA with spontaneous lower heart rate.

The main argument of using RV lead for CRT-P that the variability of AV conduction is not valid as variability in AV conduction is small [16] and almost non-existing in patients with beta-blockers in recommended doses for HF [17]. Moreover AV variability affect the percentage of LV depolarization through LV lead in the same manner, no matter the presence of RV lead. Therefore there is a need in new algorithm in DDD pacemakers able to adjust SAV/PAV intervals automatically correlated with AS-VS variability (with spontaneous variability of PR interval) in order to obtain stable LV pacing (dynamic LV stimulation). The only algorithm tested in DDD LV only patients without RV lead was auto capture in a study published in Europace by Boriani group [7].

Last but not least, after a controversial Danish trial [9], the *ICD* benefit in primary prophylaxis in patients with non-ischemic cardiomyopathy is debatable in the presence of CRT. In most European countries the majority of centres did not implant implantable cardioverter defibrillators for primary prevention despite current guideline shifting the indications toward a moderate trend from this the point of view, according to European Heart Rhythm Association Survey [18]. CRT-P indication may increase while RV pacing indication is at least debatable (where to pace, why pacing, less pacing). Systolic LV function improvement and reverse remodelling in resynchronized patients may lead to a decreased risk of sudden cardiac death and the decision of receiving a CRT-D device without experienced clinically ventricular relevant arrhythmias may lead to a device replacement dilemma [19]. In a large prospective multicentre cohort study [20] at 2 year follow-up, CRT-P patients had a 2-fold higher mortality but the cause of death analysis stated that the excess was related to non-sudden cardiac death concluding that there is considerable room for CRT-P and patients potentially may not benefit from the addition of CRT-D.

There is obvious advantage as a simpler device RA-LV only lead allows possibility of easy upgrading to CRT-D in case of secondary prevention. Due to overall result in literature, converged without study, we can speculate that validation of simpler DDD RA/LV in carefully selected CRT population may be a current trend. It may save costs, device energy and perhaps allow less complication with at least noninferiority to classic biventricular pacing.

Finally, why previous CRT-P studies did not confirmed better results in the absence of RV lead? First of all initial studies were done in sicker patients. Since then only studies with triple chamber pacemakers were done, to prove only noninferiority of LV only. To the best of our knowledge the largest DDD true LV only group was published in 2004 by Blanc JJ et al. [8]. The authors reported a 12 months follow up in this group with 10 out of 22 patients were in NYHA class IV, 7 ischemic aetiology. In this study LVEF was 21.8 ± 7.7%, LVEDD was 76.5 ± 9.4 mm and QRS duration was 182 ± 22 ms. In our group we did not implanted any NYHA IV patients, the LVEDD was significantly lower, QRS thinner and no ischemic heart disease. So the group was obviously different. Obvious we can expect better outcome. 

In a recent review prepared on behalf of the EHRA Education Committee, H. Burri et al. [21] concluded that LV univentricular pacing may lead to a better CRT outcome and may decrease the number of non-responders; to achieve this goal, the authors stated that the algorithms should automatically update AV intervals to accommodate changes in AV delay. We can emphasize after this study the real need for a better definition of who need what kind of specific CRT-P versus CRT-D tailored pacing in the real life. Not only the guideline may be improved but there is a real need in avoiding overtreatment of patients by RV lead. 

The risk of progression to high-degree atrioventricular block still exist in patients with left bundle branch block as showed in a study published in 2005 [22]; nevertheless the risk per year per 10,000 cases of LBBB was appreciated to 80, meaning 0.8% per year, compared to 5 cases per 10,000 in absence of BBB (0.05). This study is rather old; though published in 2005, it started in Göteborg in 1970 and included all men in the city born between 1915 and 1925 and did not include women. In our cohort we had only one patient in 55 upgraded from DDD RA/LV to biventricular classical triple chamber by implanting a RV lead. 

Our results should be considered in the context of several limitations. First, the present pilot study includes only patients NAVc with indication of CRT-P excluding candidates for CRT-D, making the candidates group already smaller. Our study stressed the need of a major randomized CRT trial in larger group of patients to compare LV only without RV lead versus LV only with RV lead versus true biventricular LV/RV pacing. In this way, new data would add information to the guidelines to ensure homogeneity in programming of CRT devices. Lack of long term follow-up in our study may be another limitation.

## 5. Conclusions

CRT-P using only RA/LV pacing showed a positive outcome in carefully selected patients. Cost benefit analysis, randomization and evaluation for rehospitalization, arrhythmias and complications in long term follow-up may be done in larger multicentre studies. 

## Figures and Tables

**Table 1:** Baseline demographic data.

			All Patients (*N* = 55)
Mean age (years)			62 ± 11
Male, *n* (%)			30 (55%)
NYHA functional class, *n* (%)		II	24 (44%)
		III	31 (56%)
Hypertension, *n* (%)			20 (36%)
Diabetes mellitus, *n* (%)			20 (36%)
Chronic kidney disease, *n* (%) *			23 (42%)
Medication	Beta blockers, *n* (%)		45 (82%)
	Ivabradine, *n* (%)		24 (44%)
	ACEI/ARB, *n* (%)		49 (89%)
	Diuretics, *n* (%)		52 (95%)
	Antialdosteronics, *n* (%)		47 (85%)
	Sacubitril, *n* (%)		5 (9%)

* Chronic kidney disease defined as reduction in creatinine clearance <90 mL/min. None of the patients in our cohort had creatinine clearance <30 mL/min. ACEI: angiotensin converting enzyme inhibitor; ARB: angiotensin receptor blockers.

**Table 2:** Baseline echocardiographic parameters.

Basic Echocardiographic Parameters	All Patients (*N* = 55)		All Patients (*N* = 55)	
	Mean ± SD Range		Asynchronism Parameters	
IVS (mm) LVEDD (cm) LVEDV (mL) LVESV (mL) LVEF (%) LAV (mL) sPAP (mmHg)	11.7 ± 1.6 6.4 ± 0.9 243.2 ± 82 182.4 ± 73 27 ± 5.2 104.9 ± 34 46.6 ± 15	0.9–16 4.9–8.9 110–480 80–400 15–35 50–160 20–75	Septal flash, *n* (%)	
			44 (80%)	
			Atrioventricular asynchronism, *n* (%)	
			36 (65%)	
			Intraventricular asynchronism, *n* (%)	
			41 (75%)	
			Interventricular asynchronism, *n* (%)	
			41 (73%)	
Valvulopathies	mild		moderate	severe
Mitral regurgitation	6 (11%)		27 (49%)	22 (40%)
Tricuspid regurgitation	21 (38%)		25 (46%)	9 (16%)
Aortic stenosis	1 (2%)		1 (2%)	0
Aortic regurgitation	4 (7%)		3 (5%)	0

IVS: interventricular septum thickness; LAV: left atrial volume; LVEF: left ventricular ejection fraction; LVEDD: left ventricular end-diastolic diameter; LVEDV: left ventricular end-diastolic volume; LVESV: left ventricular end-systolic volume; SD: standard deviation; sPAP: systolic pulmonary artery pressure.

**Table 3:** Electrical and echocardiographic parameters at baseline and after cardiac resynchronization therapy.

	Before RA/LV CRT-P	Mid Term Follow-Up	Relative Change *	*p* Value
QRS interval (ms), mean ± SD	164 ± 18	131 ± 7	20%	<0.0001
QRS axis (degree), mean ± SD	−17 ± 36	+37 ± 103	-	0.0006
LVEF %, mean ± SD	27 ± 5.2	37 ± 7.9	27%	<0.0001
sPAP (mmHg), mean ± SD	46.6 ± 15	41.3 ± 15	11%	0.0774
LVEDV (mL), mean ± SD	243.2 ± 82	193.7 ± 81	20%	0.0028
LVESV (mL), mean ± SD	182.4 ± 73	113 ± 63	38%	<0.0001

* value of relative change calculated as percentage. LVEF: left ventricular ejection fraction; LVEDV: left ventricular end-diastolic volume; LVESV: left ventricular end-systolic volume; SD: standard deviation; sPAP: systolic pulmonary artery pressure.
